# The Microscope

**Published:** 1853-10

**Authors:** O. W. Holmes


*The Microscope.* By O. W. Holmes.—The microscope is of all philosophical instruments the most unfailing and untiring companion. The astronomer tells us that hardly more than a dozen nights in a year are adapted to his observations. He must watch all night exposed to cold and damp, surrounded by costly and cumbrous machinery. The microscopist sits down at his fireside or his window, with a little instrument before him, a mere toy to look at —a giant mightier than the slave of the lamp or the ring in its power of transformatiion. All that he wishes to observe upon, nature is ready to furnish him. Nothing is too precious or rare for him to covet; he wishes but a mere speck, a particle, such as the koh-i-noor could spare him. Nothing is repulsive, examined in its infinitesimal shape. The disease which infected the wards of a hospital does not betray itself in the narrow apartment where he studies all its intimate details. He may study and work until *practice* comes and takes him off his feet and floats him away into a world of other cares and duties, and year after year, every day will bring him something new to examine. I will say nothing of the utlity, even the necessity of the microscope to the practical physician and the surgeon. As a mere illustrative companion to scientific study, as a mere intelligent plaything, it is the most precious gift to all who love to look at the universe as its inner life is revealed to the senses. To all who have done and are doing anything to render it more available for the purposes of study, we are under obligations which it is a pleasure to express, even if it is done as in this slight notice, which was suggested by the pleasure derived from examining the preparations made by Dr. Durkee.—*Boston Med. and Surg. Journal.*

